# *Pseudomonas* plecoglossicida infection induces neutrophil autophagy-driven NETosis in large yellow croaker *Larimichthys crocea*

**DOI:** 10.3389/fimmu.2024.1521080

**Published:** 2024-12-23

**Authors:** Jia-Feng Cao, Jiong Chen

**Affiliations:** ^1^ State Key Laboratory for Managing Biotic and Chemical Threats to the Quality and Safety of Agro-Products, School of Marine Sciences, Ningbo University, Ningbo, China; ^2^ Laboratory of Biochemistry and Molecular Biology, School of Marine Sciences, Ningbo University, Ningbo, China; ^3^ Key Laboratory of Aquacultural Biotechnology of Ministry of Education, Ningbo University, Ningbo, China

**Keywords:** *Pseudomonas plecoglossicida*, *Larimichthys crocea*, neutrophil extracellular traps, autophagy, antibacterial

## Abstract

Neutrophil extracellular traps (NETs) are crucial for the immune defense of many organisms, serving as a potent mechanism for neutrophils to capture and eliminate extracellular pathogens. While NETosis and its antimicrobial mechanisms have been well studied in mammals, research on NETs formation in teleost fish remains limited. In this study, we used the large yellow croaker (*Larimichthys crocea*) as the study model to investigate NETosis and its role in pathogen defense. Our results showed that infection with *Pseudomonas plecoglossicida* could induce NETosis. To further explore the underlying mechanism, we performed transcriptome analysis and western blotting, which revealed that *P. plecoglossicida* triggers NETosis through activation of the autophagy pathway. Inhibition of autophagy significantly reduced NET production, highlighting its critical role in this process. Furthermore, our studies demonstrated that NETs exert a bacteriostatic effect, significantly suppressing the growth of *P. plecoglossicida*. Taken together, our findings reveal that autophagy regulates NETosis in large yellow croaker and underscore the essential role of NETs in bacterial defense, providing new insights into immune responses in teleost fish.

## Introduction

Neutrophils, which are polymorphonuclear and granular leukocytes, constitute 50%-70% of all circulating leukocytes in humans and play a critical role in the innate immune system (1). In mammals, there cells are primarily generated in the bone marrow and subsequently released into the bloodstream, where they can rapidly migrate to infection or inflammation sites via the leukocyte adhesion cascade (2, 3). Upon reaching the affected area, neutrophils initiate powerful antimicrobial responses as the first line of defense against pathogens (4–6). These responses include degranulation (7), the generation of reactive oxygen species (ROS) (8–10), the initiation of phagocytosis (11), and the formation of the neutrophil extracellular traps (NETs) (12, 13). Ultimately, these processes, often in collaboration with macrophages, contribute to the effective elimination of pathogens (14).

NETs are fibrous structures composed of chromatin that is condensed and decorated with histones, antimicrobial proteins, and enzymes derived from neutrophil granules (15, 16). First described by Brinkmann et al. in 2004 (17), NETs have become a focal point in the study of neutrophil immune functions. The formation of NETs, or NETosis, can occur in two main forms: suicidal, where neutrophils undergo programmed cell death during NETs release; and vital, where neutrophils remain active while releasing NETs. Both forms of NETs can capture and kill a wide variety of pathogens, including bacteria, viruses, fungi, and parasites (18–20). In mammals, NETs formation can be triggered by various of exogenous stimuli, such as phorbol 12-myristate 13-acetate (PMA), lipopolysaccharides (LPS), as well as fungi, parasites, bacteria, and viruses (21, 22). Moreover, regulatory mechanisms controlling NETosis have also been identified. For example, activation of autophagy (via inhibition of the mTOR pathway or activation of cGAS-STING signaling pathway) promotes the formation of NETs (23, 24), whereas inhibition of autophagy (use PtdIns3K inhibitor) reduces the release of NETs (25), suggesting that autophagy is essential for NETosis. Research on NETs extends beyond mammals to teleost fish species, including the fathead minnow (*Pimephales promelas*) (26), zebrafish (*Danio rerio*) (27), common carp (*Cyprinus carpio*) (28), turbot (*Scophthalmus maximus*) (29, 30), tongue sole (*Cynoglossus semilaevis*) (31), and rainbow trout (*Oncorhynchus mykiss*) (32) among other teleost fish. In turbot, NETs can be induced *in vitro* by *Edwardsiella piscicida* (*E. piscicida*) and have been shown to trap and kill bacteria (29). Similarly, in tongue sole, NETosis can be triggered by *Pseudomonas fluorescens* (*P. fluorescens*) and *Vibrio harveyi* (*V. harveyi*), with the resulting NETs exhibiting bactericidal activity (30). Despite these findings, the mechanisms underlying NETosis in teleost fish remain less well understood compared to mammals, particularly with respect to the role of autophagy in regulating this process. Given the evolutionarily conservation of autophagy, it is crucial to understand whether and how autophagy signaling regulates NETosis in teleost fish models.

The large yellow croaker (*Larimichthys crocea*) is a commercially important species in China’s marine aquaculture industry. However, bacterial infections, especially visceral white spot disease (VDPD) caused by *Pseudomonas plecoglossicida* (*P. plecoglossicida*), pose significant challenges to the industry’s growth (33, 34). *P. plecoglossicida* is a pathogen not only of the large yellow croaker but also of other farmed fish species, such as *Epinephelus coioides* and *Plecoglossus altivelis*, and is equally pathogenic (35, 36), where it induces inflammation and cell death (37). In this study, we used the large yellow croaker as a model to investigate the regulatory mechanisms of NETosis. We observed that *P. plecoglossicida* infection induced both autophagy and NETosis in neutrophils. Notably, the autophagy pathway was activated in neutrophils upon infection, and inhibition of autophagy was found to significantly reduce the formation of NETs. Furthermore, we confirmed the antibacterial properties of the NETs produced in response to *P. plecoglossicida*. In summary, our findings provide new insights into the regulatory role of autophagy in NETosis of teleost fish. This study contributes to a better understanding of immune responses in aquaculture and highlights the potential of targeting autophagy pathways to enhance fish immune defenses against bacterial infections.

## Materials and methods

### Experimental animals

Healthy large yellow croaker (*Larimichthys crocea*) (~60g) were sourced from a croaker farming base in Xiangshan harbor (Zhejiang, China) and then maintained in a recirculating aquatic system at Ningbo University for a period exceeding 2 weeks prior to conducting the experiment. The fish were kept at 22°C and fed daily with commercial diet. Feeding was terminated 48 h prior to the sampling experiment. The entire animal husbandry practices and experimental methodologies were approved by Institutional Animal Care and Use Committee of Ningbo University (permit number 12841).

### Neutrophil isolation

To gain neutrophils, we first sampled the large yellow croaker head kidney, and the head kidney was treated with DMEM (supplemented with 5% FBS) and mechanically disaggregated on a 100-μm nylon cell strainers (BD Biosciences, Franklin Lakes, USA). Then, the obtained cell suspensions were placed into 61% (1.080g/mL) to 72% (1.094g/mL) percoll (GE Healthcare, Chalfont St. Giles, UK) discontinuous density gradient and centrifuged at 400 g for 30 min at 4°C. The neutrophils at the interface were collected and washed with DMEM (supplemented with 5% FBS). Subsequently, the purity of the collected neutrophils was verified by flow cytometry SSC and FSC, and the category of granulocytes was verified by using Giemsa staining according to the instructions, and the high purity neutrophils obtained were pending further analysis.

### RNA isolation and quantitative real-time PCR analysis

Total RNA from neutrophils was extracted with TRIzol Reagent (Invitrogen, Carlsbad, USA) according to the manufacturer’s protocol. Briefly, the purity and concentration of the extracted RNA was carried out by spectrophotometry (NanoPhotometer NP80 Touch), and the integrity of the RNA was determined by agarose gel electrophoresis. The quantified RNA samples were then used for cDNA synthesis (Invitrogen, Carlsbad, CA, USA). Thereafter, qPCR was performed on a 7,500 qPCR system (Applied Biosystems) with the cDNA using the EvaGreen 2 × qPCR Master mix (Yeasen, Shanghai, China). All samples were performed following conditions: 95°C for 5 min, followed by 38 cycles at 95°C for 10 s and at 60°C for 30 s. A dissociation protocol was carried out after thermos cycling to confirm only one amplicon of each gene. β-actin was used as the housekeeping gene for the normalization of target gene expression. The relative expression level of all target genes was determined using the Pfaffl method (38). The primer sequences used for qPCR are listed in **Supplementary Table S3**.

### Sequencing and analyses

The neutrophil samples of the control group and the infected group were sent to Wuhan Benagen Technologies Co., Ltd. (Wuhan, China). Briefly, the RNA was extracted using the Total RNA Kit I, and the concentration and integrity of RNA were checked by NanoDrop One spectrophotometer (NanoDrop Technologies, Wilmington, DE) and Agilent 2100 Bioanalyzer, respectively. Thereafter, the RNA was used for stranded RNA sequencing library preparation using a MGIEasy RNA Library Prep Kit for BGI^®^ following the manufacturer’s instruction. PCR products underwent enrichment, quantification procedures, and ultimately underwent sequencing using the DNBSEQ. The clean reads were aligned to the genome of the large yellow croaker. Use gffcompare program to compare merged transcripts with known transcripts of the genome, discovering new transcripts and new genes to complement existing annotations. Differential expression analysis of two groups was performed using the DESeq2. The FDR ≤ 0.05 and Fold Change ≥ 2 was set as the threshold for significantly differential expression. For further analysis of the DEGs, we carried out a KEGG enrichment using kofam_scan software to identify the major pathways that were significantly enriched after *P. plecoglossicida* treatment.

### SDS-PAGE and western blot

The neutrophil intracellular proteins were resolved on 15% SDS-PAGE gel (Bio-Rad, Hercules, USA) under reducing conditions, and the gels were transferred onto a 0.22 μm PVDF membrane and blocked in 0.05% PBST with 5% skim milk. Then, the membrane was incubated with anti-p62/SQSTM1, anti-LC3B polyclonal rabbit antibody (Servicebio, Wuhan, China), or anti-β-actin monoclonal rabbit antibody (ABclonal Technology, Wuhan, China) for 1 h on a shaker. After washing four times with 0.05% PBST (5 min/time), goat anti-rabbit IgG was added and incubated for 45 min at room temperature. After washing four times with 0.05% PBST (5 min/time), the reaction bands were visualized with the ECL Substrate (Bio-Rad, Hercules, USA) and then scanned by Tanon-5200 Chemiluminescent Imaging System (Tanon Science & Technology). The densitometry of target band was analyzed with ImageJ software.

### Immunofluorescence microscopy

The microscopic observation of NETs was consistent with that previously reported (29). Briefly, *P. plecoglossicida* was cultured in TSB medium to an OD_600_ of 0.8 at 26°C. Neutrophils (~2 × 10^5^) were inoculated onto glass slides that had been treated with 0.001% polylysine (Sigma, St. Louis, MO, USA) and placed in a 24-well cell culture plates. Cells were left to rest for 2 h and then treated with *P. plecoglossicida* (~ 10^6^ CFU) at 26°C for 2 and 4 h. Control cells were left untreated. Control cells were left untreated. Under a fluorescence microscope, SYTOX Green (Beyotime, Shanghai, China) was added to the cells, and after incubation for 5 min, the cells were washed three times with PBS. Cells were then fixed with 4% paraformaldehyde (Sigma-Aldrich, St. Louis, USA) for 25 min and stained with 4’,6-diamidino-2-phenylindole (DAPI) (1 mg/ml; Invitrogen, Carlsbad, USA) according to the manufacturer’s instructions. Scanning electron microscopy (SEM) was performed as reported (39).

### Autophagy assay

To detect the percentage of autophagy of neutrophils after *P. plecoglossicida* treatment. According to the manufacturer’s instructions with sight modifications, briefly, the large yellow croaker neutrophils (3 × 10^6^ cells/well in a volume of 200 μL) were collected after treatment with *P. plecoglossicida* (1 × 10^6^ CFU) for 1, 2, and 4 h or pre-treatment with autophagy inhibitor 3-methyladenine (3-MA) (GlpBio, Wuhan, China) for 1 h and then with *P. plecoglossicida* (1 × 10^6^ CFU) for 1, 2, and 4 h. Control cells were untreated. MDC (Beyotime, Shanghai, China) were added to the neutrophils and incubated for 20 min at 26°C and analyzed using a fluorescence microplate reader (Molecular Devices, Sunnyvale, USA) at 360 nm excitation and 512 nm emission and expressed as relative fluorescence units (RFU).

### Proliferation of *P. plecoglossicida* captured by NETs

To investigate the effect of NETs on the proliferation of *P. plecoglossicida*, we examined the survival of bacteria captured by NETs. Briefly, *P. plecoglossicida* was cultured in TSB medium to an OD_600_ of 0.8 at 26°C. Bacteria were collected by centrifugation at 14,000 *g* and washed three times with PBS and resuspended. Neutrophils (~2 × 10^5^ cells/well in a volume of 200 μL) were seeded in a 96-well cell culture plate and stimulated with PMA (1 μg/mL) at 26°C for 2 h. The cells were centrifuged at 400 g for 5 min, and 150 μL of the supernatant was discarded. 50 μL of DMEM medium with or without 100 U/ml DNase I was then added to the cells. *P. plecoglossicida* (~10^4^ CFU) was then added to the culture plates. centrifugation at 800 g for 10 min brought the bacteria into close contact with the neutrophils. The plates were then incubated at 26°C for 1, 3, and 6 h. At the end of the incubation, the contents of each well (bacteria + neutrophils and NETs) were collected and serially diluted, and one part of the diluted contents was plated on TSA agar plates to observe subsequent colony formation, and the other part was seeded into TSB medium for incubation and the OD values were determined spectrophotometrically at 2, 4, 6, 8, 10, 12, 14, and 16 h after incubation, respectively.

### Quantification of NETs

To quantify NETs, we used the method of Parker et al. (40). Briefly, neutrophils (3 × 10^6^ cells/well in a volume of 200 μL) were suspended in HBSS and inoculated in black 96-well plates. Cells were treated with *P. plecoglossicida* (1 × 10^6^ CFU) for 1, 2, and 4 h or pre-treated with 3-MA for 1 h followed by *P. plecoglossicida* (1 × 10^6^ CFU) for 1, 2 and 4 h. Control cells were untreated. After treatment, SYTOX Green was added to the cells and incubated for 5 min. Fluorescence was then quantified using a fluorescence microplate reader at 488 nm excitation and 536 nm emission and expressed as RFU.

### Statistical analysis

Statistical evaluations were performed using GraphPad Prism 9. Data comparisons between groups were determined by Student’s *t* test. Data were expressed as the mean ± SEM, *p* < 0.05 was considered statistically significant.

## Results

### Large yellow croaker NETosis induced by *P. plecoglossicida*

**Figure 1:**
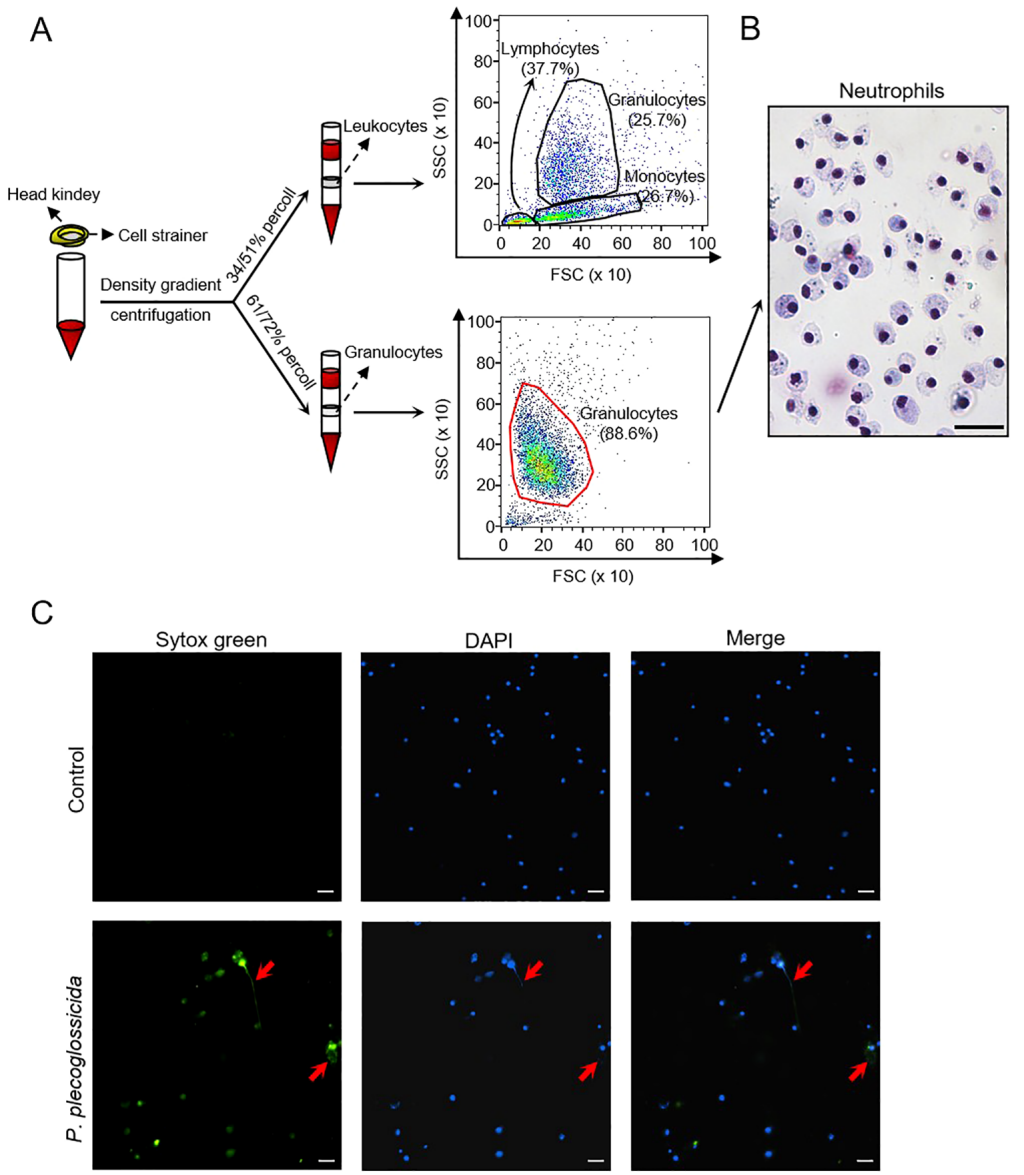
Isolation and identification of large yellow croaker neutrophils and the effect of *P. plecoglossicida* on the NETs formation. **(A)** Isolation of leucocytes and neutrophils from the head kidney and validation by flow cytometry. **(B)** Giemsa staining of neutrophils. Scale bar, 20 μm. **(C)** Neutrophils in the control and *P. plecoglossicida* groups (*P. plecoglossicida* incubated with neutrophils for 2 h) were stained with SYTOX Green and DAPI and analyzed by immunofluorescence microscopy. Arrows indicate NETs, Scale bar, 20 μm.

To investigate the impact of *P. plecoglossicida* on neutrophils in large yellow croaker, we isolated head kidney granulocytes using a percoll discontinuous density gradient (55/61%) separation method. Flow cytometry analysis revealed that the high purity cell population (88.6%) we obtained closely matched the granulocyte distribution within the head kidney leukocytes, indicating that the isolated cells were predominantly granulocytes (**Figure 1A**). To confirm the identity of these cells as neutrophils, we conducted Giemsa staining, which showed that the cytoplasm of these cells was predominantly stained mauve (neutrophils), rather than light red (eosinophils) or purple (basophils), further supporting that the isolated granulocytes were primarily neutrophils (**Figure 1B**). Subsequently, we co-incubated *P. plecoglossicida* with neutrophils for 2 and 4 h, respectively, and observed the formation of NETs using immunofluorescence microscopy (**Figure 1C**; **Supplementary Figure S1**). This confirmed our hypothesis that *P. plecoglossicida* induces NETosis in neutrophils.

### Analysis of transcriptomic changes in large yellow croaker neutrophils after *in vitro P. plecoglossicida* treatment

To elucidate the mechanisms underlying the induction of NETosis by *P. plecoglossicida*, we constructed RNA-Seq libraries from six samples, comprising three controls (LiC group) and three infected with *P. plecoglossicida* (LiE group). These libraries were sequenced using the Illumina platform. Following quality filtering, we obtained a total of 257,097,340 clean reads (**Supplementary Table S1**). Subsequently, these clean reads were mapped back to the *Larimichthys crocea* genome, with each sample achieving a mapping rate exceeding 90% (**Supplementary Table S2**).

**Figure 2:**
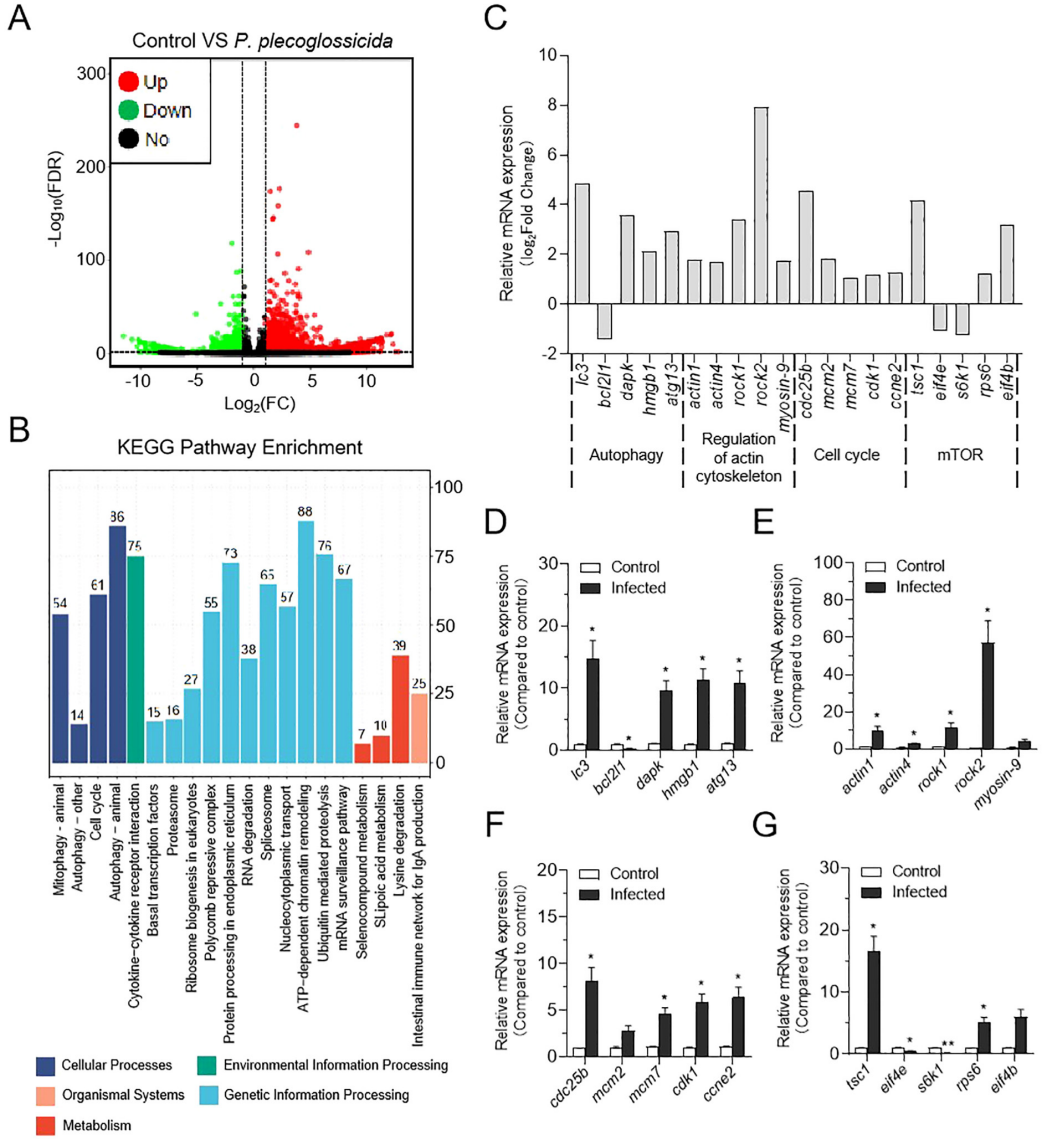
Effect of *P. plecoglossicida* infection on transcriptome changes in neutrophils of the large yellow croaker. **(A)** Volcano plot showing the distribution of DEGs in the *P. plecoglossicida* infection group compared to the control group. **(B)** Counts of DEGs in each KEGG pathway. Different colors mean different classes. **(C)** The expression of major genes in the autophagy, regulatory actin cytoskeleton, cell cycle, and mTOR pathways in RNA sequencing. **(D–G)** qPCR validation of DEGs in the pathways of autophagy, regulation of the actin cytoskeleton, cell cycle, and mTOR (*n* = 3 per group). Statistical differences were performed by Student’s *t*-test. Data in **(D–G)** are representative of at least three independent experiments (Mean ± SEM). *P < 0.05, **P < 0.01.

Through statistical analysis, 1,577 differential expressed genes (DEGs) were identified in the LiE/LiC group (618 up-regulated and 959 down-regulated). The expression pattern of DEGs was presented in the volcano plots (**Figure 2A**). To further investigate the changes in DEGs after *P. plecoglossicida* treatment of neutrophils, we performed Gene Ontoloy (GO) enrichment (biological process) and KEGG pathway enrichment analysis. As illustrated in **Supplementary Figure S2**, metabolic processes were notably enriched, suggesting that cellular vital activities and nutrient metabolism were significantly impacted by *P. plecoglossicida* infection. In KEGG pathway enrichment analysis, numerous essential signaling pathways were successfully identified. The results indicated enrichment in pathways integral to neutrophil life activities, including autophagy (closely associated with the formation of NETs), ubiquitin mediated proteolysis (a critical protein degradation mechanism within the cell that plays a pivotal role in cell biological processes), mitophagy, ribosome biogenesis in eukaryotes, and the cell cycle (relevant to the regulation of cell proliferation post-bacterial infection) (**Figure 2B**). Despite the significant enrichment of autophagy signaling pathways, some pathways related to NETs formation were not enriched, such as regulation of actin cytoskeleton and mTOR signaling pathways, which have been well-demonstrated in mammals (24, 41–43). Consequently, we examined these pathways in the transcriptome data and found that regulation of actin cytoskeleton is promoted following *P. plecoglossicida* infection, whereas the mTOR pathway is suppressed (**Figure 2C**). To validate the RNA-Seq expression levels, we detected the expression levels of the corresponding genes in **Figure 2C** using qRT-PCR. The relative mRNA expression levels determined by RNA-Seq and qRT-PCR were significantly correlated (**Figures 2C–G**), demonstrating that RNA-Seq and qRT-PCR possess comparable sensitivity and accuracy for assessing gene expression *in vitro*.

### *P. Plecoglossicida* induces autophagy in large yellow croaker neutrophils

**Figure 3:**
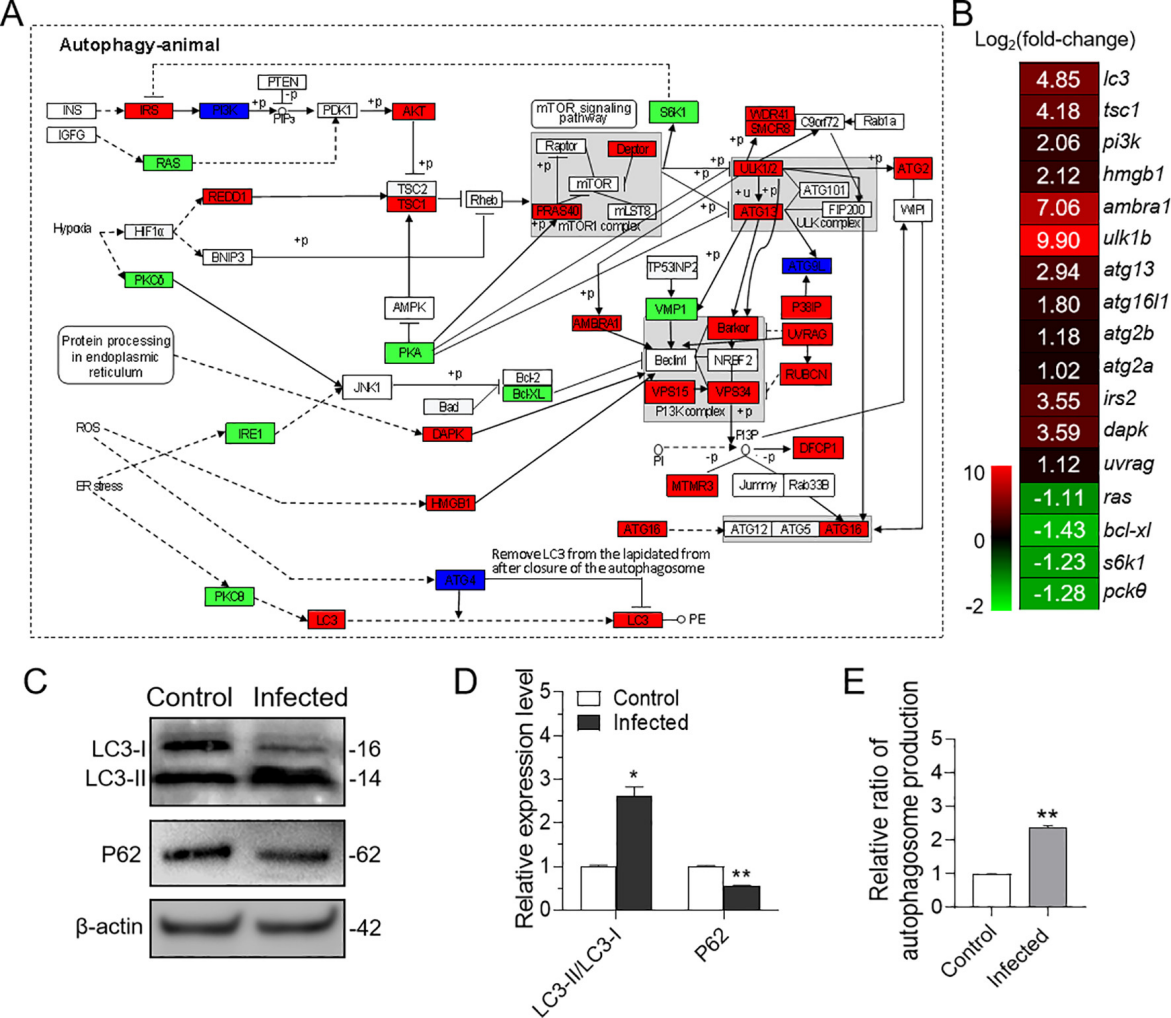
Effect of *in vitro* infection of neutrophils with *P. plecoglossicida* on autophagy. **(A)** Both of red, green, and deep blue shading boxes represent molecules of the autophagy pathway identified in head kidney neutrophils of large yellow croaker. And the red boxes indicate the up-regulated DEGs in this pathway, the green boxes indicate the down-regulated DEGs in this pathway, and the deep blue boxes indicate both up-regulated and down-regulated DEGs in this pathway. **(B)** Differential expression genes involved in the autophagy pathway were analyzed after *P. plecoglossicida* infection. The color gradient represents highly up-regulated (red) to highly down-regulated (green) genes. **(C)** Immunoblot analysis of LC3 and p62/SQSTM1 protein levels in neutrophils infected with *P. plecoglossicida* for 2 h. **(D)** The relative proportions of LC3-II/LC3-I and p62/SQSTM1 in the infected compared with the control group were evaluated by densitometric analysis of immunoblots from **(C)** (*n* = 3 per group). **(E)** Neutrophils were infected with or without *P. plecoglossicida* for 2 h, and autophagy fluorescence intensity was detected (*n* = 3 per group). Statistical differences were performed by Student’s *t*-test. Data in **(D, E)** are representative of at least three independent experiments (Mean ± SEM). *P < 0.05, **P < 0.01.

In mammals, autophagy has been implicated in the induction of NETosis. To investigate whether a similar process occurs in teleost fish, we examined the transcriptome of neutrophils infected with *P. plecoglossicida.* Our analysis revealed that the autophagy signaling pathway was activated upon infection (**Figure 3A**), and several key genes within this pathway were significantly up-regulated (**Figure 3B**). These transcriptomic findings led us to hypothesize that *P. plecoglossicida* infection could trigger autophagy in neutrophils. To substantiate this speculation, we conducted further analyses at the protein and cellular levels. Western blot results demonstrated that the expression of p62/SQSTM1 protein was markedly reduced, and the ratio of LC3-II/LC3-I was significantly elevated in neutrophils from the infected group compared to the control group (**Figures 3C, D**). Additionally, fluorescence microplate reader results also confirmed that neutrophils exhibited more pronounced autophagy following infection (**Figure 3E**). Collectively, these results substantiate the induction of autophagy in neutrophil by *P. plecoglossicida*.

### Autophagy regulates NETosis in large yellow croaker

**Figure 4:**
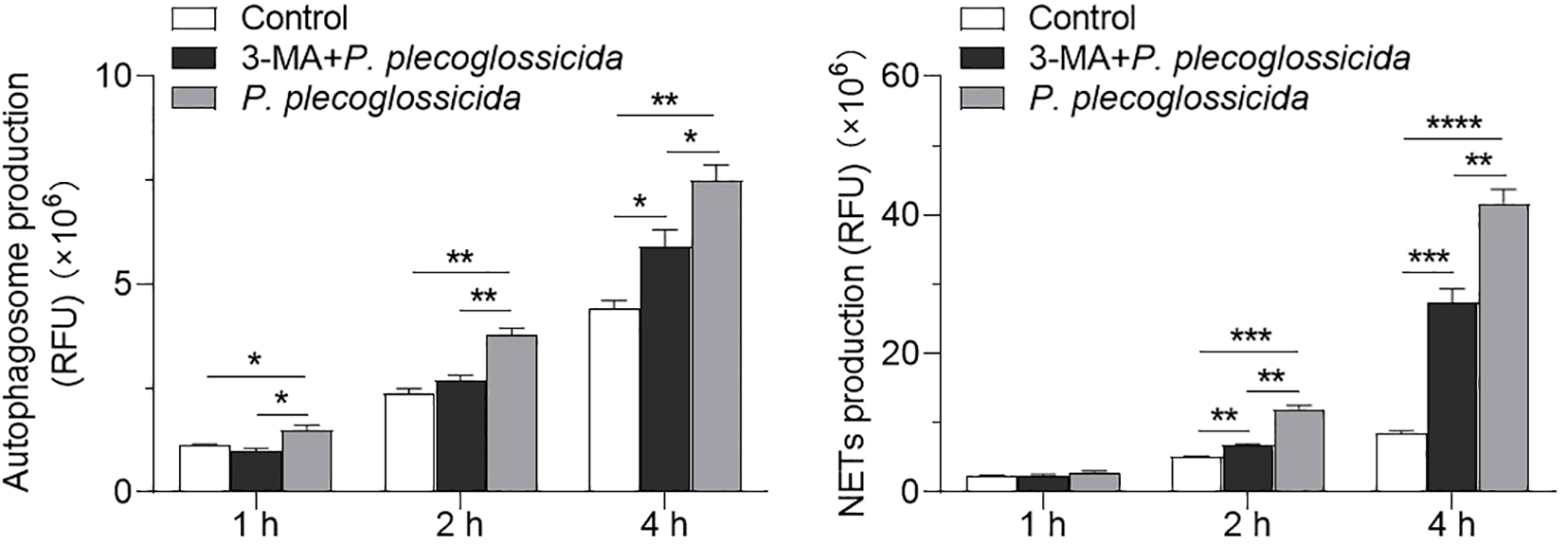
Effect of the inhibition of autophagy on the NETs formation in neutrophils. **(A)** Large yellow croaker neutrophils were treated with or without 3-MA and then infected with *P. plecoglossicida* for different times to detect autophagy production. The control group was untreated (*n* = 3 per group). **(B)** Large yellow croaker neutrophils were treated with or without 3-MA and then infected with *P. plecoglossicida* for different times to detect NETs production. The control group was untreated (*n* = 3 per group). Statistical differences were performed by Student’s *t*-test. Data in **(A, B)** are representative of at least three independent experiments (Mean ± SEM). *P < 0.05, **P < 0.01, ***P < 0.001, ****P < 0.0001.

To explore the role of autophagy in regulating NETosis, we established a model of autophagy inhibition using 3-MA. Fluorescence microplate reader analysis revealed that neutrophils pretreated with 3-MA exhibited a significant decrease in autophagy levels following infection with *P. plecoglossicida* at 1, 2, and 4 h post-infection, as compared to the infected group (**Figure 4A**). Subsequently, we further validated the impact of autophagy on NETosis through similar fluorescence microplate reader assays. The results indicated that the formation of NETs was notably reduced in neutrophils pretreated with 3-MA and then infected, at both 2 and 4 h post-infection, relative to the infected group. Meanwhile, the infected group demonstrated a significant increase in NETs formation compared to the control group (**Figure 4B**). Collectively, these findings underscore the critical role of autophagy in the NETosis process, wherein activation of autophagy enhances NETosis, whereas inhibition of autophagy dampens it.

### NETs possess the capacity to inhibit the proliferation of *P. plecoglossicida*

**Figure 5:**
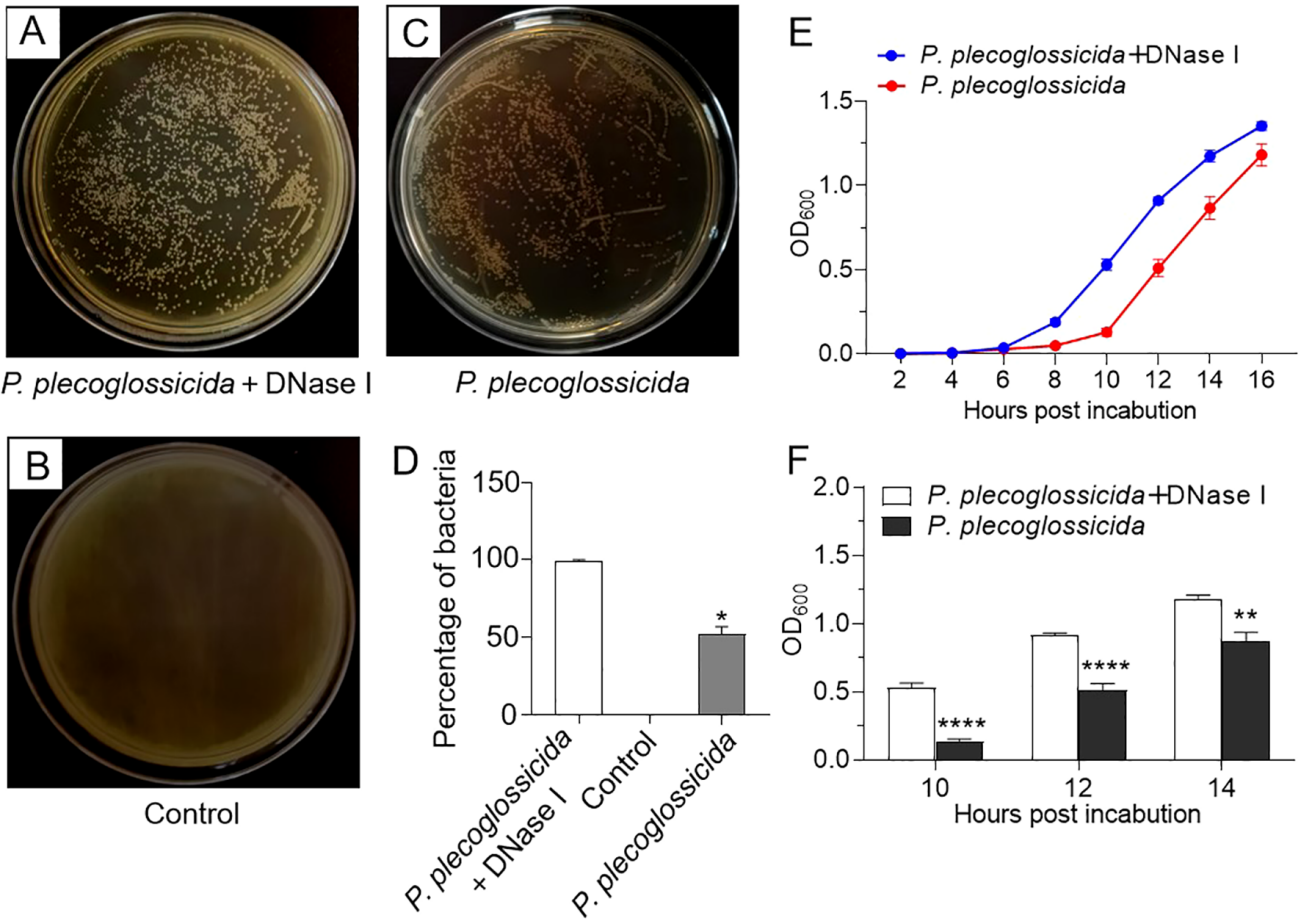
Effects of neutrophil NETs on survival and growth of *P. plecoglossicida*. **(A–C)** Large yellow croaker neutrophils were pretreated with PMA for 2h and then infected with *P. plecoglossicida* before being treated with or without DNase I for 6 h, and the collected mixtures (bacteria + neutrophils and NETs) were inoculated into the TSA agar plates to observe colony formation. The control group was untreated. **(D)** Graph showing the number of colonies in **(A–C)** (*n* = 3 per group). **(E)** Large yellow croaker neutrophils were infected with *P. plecoglossicida* and then treated with or without DNase I for 6 h, and the collected mixtures (bacteria + neutrophils and NETs) were inoculated into the TSB medium to observe the OD value of the *P. plecoglossicida* growth curve (*n* = 3 per group). **(F)** Statistical plots of OD values at 10, 12, and 14 h in F (*n* = 3 per group). Statistical differences were performed by Student’s *t*-test. Data in **(D–F)** are representative of at least three independent experiments (Mean ± SEM). *P < 0.05, **P < 0.01, ****P < 0.0001.

In order to investigate the impact of NETs on bacterial growth, we conducted growth experiments. The results showed that compared with *P. plecoglossicida* infection group (NETs-positive group), the *P. plecoglossicida* + DNase I group (NETs-negative group) exhibited a greater number of colony formation on TSA agar plates following incubation with neutrophils (**Figures 5A–D**). Additionally, the NETs-negative group demonstrated a more rapid growth rate of *P. plecoglossicida* in TSB medium compared to the NETs-positive group (**Figures 5E, F**). These findings confirm that neutrophil NETs are capable of capturing and suppressing the growth of *P. plecoglossicida*, emphasizing their significant role in the resistance to bacterial invasion.

## Discussion

Neutrophils are the first line of defense in innate immunity, rapidly mobilizing to sites of infection or tissue damage. Historically, their bactericidal activity have been attributed primarily to phagocytosis, where microorganisms are engulfed and subsequently destroyed within phagosomes by various proteases. Additionally, neutrophils release antimicrobial proteins from their granules, which can act both intracellularly on phagosomes and extracellularly to eliminate pathogens (44). However, the paradigm shifted in 2004 when Brinkmann et al. revealed that a novel mechanism: neutrophils can extrude DNA to form NETs, which have the ability to capture and kill bacteria (17). This discovery transformed our understanding of neutrophil function and sparked widespread interest in the study of NETs, particularly within mammalian models. In contrast, research on NETs in fish has lagged behind, and relatively few studies have explored the regulatory mechanisms underlying NETs formation. To date, only a handful of reports have confirmed the regulatory roles of ROS, nitric oxide (NO), myeloperoxidase (MPO), and pyroptotic signaling in NETosis (30, 31). Therefore, to expand our understanding of NETs formation in fish, we investigated the mechanism of *P. plecoglossicida*-induced NETosis in large yellow croaker.

Previous studies has shown that several aquaculture-relevant bacteria, including *E. piscicida*, *P. fluorescens*, and *V, harveyi*, can induce NETosis (30, 31). Building upon this foundation, our current study focused on the effect of *P. plecoglossicida* on large yellow croaker neutrophils. Upon bacterial stimulation, we observed that these neutrophils released filamentous, net-like structures, consistent with the formation of NETs. To further investigate this phenomenon, we used double staining with SYTOX Green (a DNA dye that easily passes through damaged cytoplasmic membranes but not through the plasma membranes of living cells) and DAPI (a DNA dye that penetrates intact cell membranes to stain the nucleus). The staining results showed that both the neutrophils and the extracellular structures were labeled, suggesting that the NETs formation in these cells was associated with progressive cell death. This observation is consistent with the phenomenon of suicidal NETosis in mammals, where the formation of NETs is often linked to cell death (45).

To further explore the potential molecular mechanisms underlying *P. plecoglossicida*-induced NETosis in large yellow croaker, we analyzed the effects of *P. plecoglossicida* on neutrophils at the transcriptome level. When the organism is infected by pathogens, a range of cellular life activities are affected, such as cell survival, death, and metabolism of substances (proteins and nucleic acids). Based on the results of the transcriptome KEGG signaling pathway and GO enrichment (biological processes) analyses, our results also revealed that a significant number of pathways associated with autophagy, cell cycle, ubiquitin mediated proteolysis, and ribosome biogenesis in eukaryotes, and metabolism-related processes were highly enriched in neutrophils. Previous research has established that autophagy plays a role in various neutrophil functions, such as differentiation, phagocytosis, cytokine production, degranulation, cell death, and NETosis. Inhibition of autophagy has been shown to prevent the formation of NETs in neutrophils (46, 47). Notably, our transcriptome and qPCR data indicated that the autophagy signaling pathway was markedly activated in neutrophils following infection with *P. plecoglossicida*, with numerous autophagy-related genes significant up-regulated. This suggests that, akin to mammals, autophagy may regulate NETosis in large yellow croaker. Additionally, studies in mammals have demonstrated that the mTOR signaling pathway and regulation of actin cytoskeleton are closely associated with the formation of NETs, and their inhibition leads to impaired NETosis (24, 41–43). Although our analysis did not show enrichment for these pathways, examination of their respective genes revealed that both the mTOR signaling pathway and actin cytoskeleton regulation were suppressed at the overall level post-infection. This finding suggests that while these pathways may not be directly enriched in our transcriptome data, they could still play a role in the regulation of NETosis in large yellow croaker, potentially in conjunction with autophagy.

Autophagy is an intracellular degradation process that transports cytoplasmic material to lysosomes or autophagic vesicles for degradation. This process is crucial in a wide range of physiological and pathological conditions across species from yeast to mammals. To measure autophagic activity, we employed immunoblotting to assess key markers of autophagy, including LC3 and p62/SQSTM1 (48). In mammals, LC3-I is conjugated to phosphatidylethanolamine (PE) to form LC3-II during autophagy, while p62/SQSTM1 levels decrease upon autophagy induction (49, 50). Western blot analysis revealed that *P. plecoglossicida* infection significantly increased the LC3-II/LC3-I ratio in neutrophils, while p62/SQSTM1 levels were notably reduced, confirming the induction of autophagy. Subsequently, to further investigate the relationship between autophagy and NETs formation, we treated neutrophils with 3-MA, a non-selective inhibitor of autophagy that blocks phosphatidylinositol 3-kinase (PI3K) activity (51, 52). In mammals, 3-MA inhibits autophagy and attenuates the release of NETs in neutrophils during Gram-negative sepsis (53). In our study, with large yellow croaker, we observed that treating neutrophils with 3-MA prior to *P. plecoglossicida* infection led to a significant decrease in both autophagy and NETs production compared to the group directly infected with *P. plecoglossicida*. This inhibition of autophagy corresponded with reduced NETs production. Intriguingly, we found that autophagy preceded NETs formation, suggesting a temporal relationship between autophagy and NETosis.

NETs have been shown to capture and eliminate various pathogens, though their effectiveness can vary depending on the pathogen and environmental conditions. For instance, mammalian NETs can trap *Streptococcus pneumoniae* and *Streptococcus aureus* without killing them (54, 55), whereas they can directly eradicate *Streptococcus flexneri* and *Candida albicans* (17, 56). A similar pattern is observed in fish, where turbot NETs can eliminate *Escherichia coli* but not *P. fluorescens* (29), and tongue sole NETs can immobilize and disrupt the replication of *V. harveyi* and *P. fluorescens* without directly killing them. Notably, NETs did not significantly impede the proliferation of *Edwardsiella tarda* (*E. tarda*), indicating that *E. tarda* may possess a mechanism to counteract the antimicrobial effects of NETs (31). In the case of large yellow croaker, we observed that NETs captured *P. plecoglossicida* and slowed its growth. After a 6-hour incubation with neutrophils, both bacterial counts and OD measurements revealed that the growth of *P. plecoglossicida* was significantly slower compared to the NETs-negative group (*P. plecoglossicida* + DNase I group). Interestingly, neutrophils incubated with *P. plecoglossicida* for 1 or 3 h did not show a significant difference compared to the NETs-negative group (data not shown). These results collectively indicate that while NETs may limit bacterial growth, they do not necessarily result in complete bacterial eradication.

**Figure 6:**
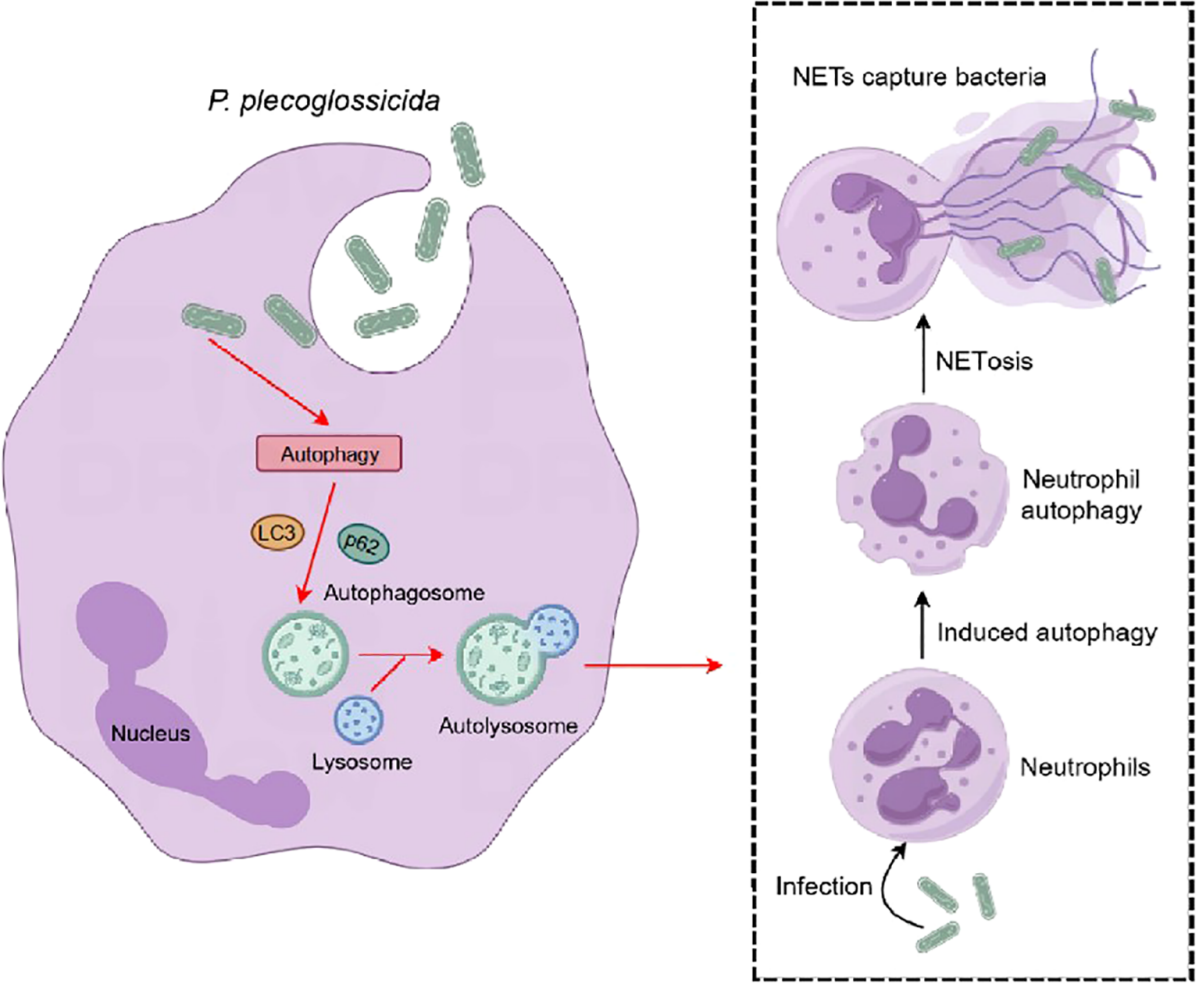
Pattern diagram of *P. plecoglossicida* induced NETosis in the large yellow croaker. After infection with *P. plecoglossicida*, the autophagy pathway is activated in large yellow croaker neutrophils, which in turn causes autophagy in neutrophils and induces NETosis, and the formation of NETs can capture the pathogenic bacterium *P. plecoglossicida* to prevent its invasion of the organism.

In conclusion, our study provides compelling evidence that autophagy signaling pathways are pivotal in regulating the formation of NETs in large yellow croaker neutrophils during infection with *P. plecoglossicida* (**Figure 6**). Our findings suggest that autophagy is essential for the process of NETosis and that NETs capture and suppressed the proliferation of *P. plecoglossicida*. These results highlight the evolutionary conservation of the autophagy signaling pathway in regulating NETs formation and offer valuable insights into the immune responses of teleost fish, with potential implications for enhancing disease resistance in aquaculture.

## Data Availability

The data presented in the study are deposited in the NCBI Bioproject repository, accession number PRJNA1197011.

## Supplementary material

Supplementary Figure 1 Effect of *P. plecoglossicida* on the formation of neutrophil NETs. Neutrophils in *P. plecoglossicida* groups (*P. plecoglossicida* incubated with neutrophils for 4 h) were stained with DAPI and analyzed by immunofluorescence microscopy. Arrows indicate NETs, Scale bar, 20 μm.

Supplementary Figure 2 Effect of *P. plecoglossicida* infection on GO Enrichment (Biological Process) in neutrophils of the large yellow croaker. The top 20 GO Enrichment (Biological Process) terms for DEGs.
