# Supplementary figures and images for: Pseudomonas plecoglossicida infection induces neutrophil autophagy-driven NETosis in large yellow croaker Larimichthys crocea
Source: Front Immunol. 2024 Dec 23;15:1521080. doi: 10.3389/fimmu.2024.1521080 (PMC11701331; doi:10.3389/fimmu.2024.1521080)

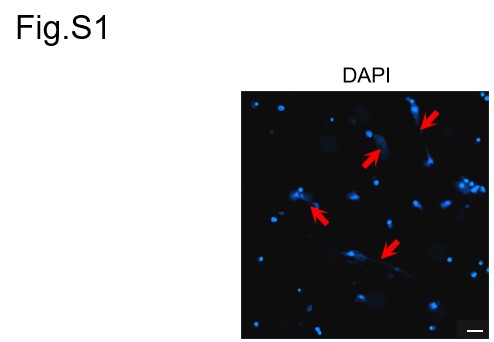

Supplement: Supplementary Figure 1 — Effect of P. plecoglossicida on the formation of neutrophil NETs. Neutrophils in P. plecoglossicida groups (P. plecoglossicida incubated with neutrophils for 4 h) were stained with DAPI and analyzed by immunofluorescence microscopy. Arrows indicate NETs, Scale bar, 20 μm. [file Image1.jpeg]

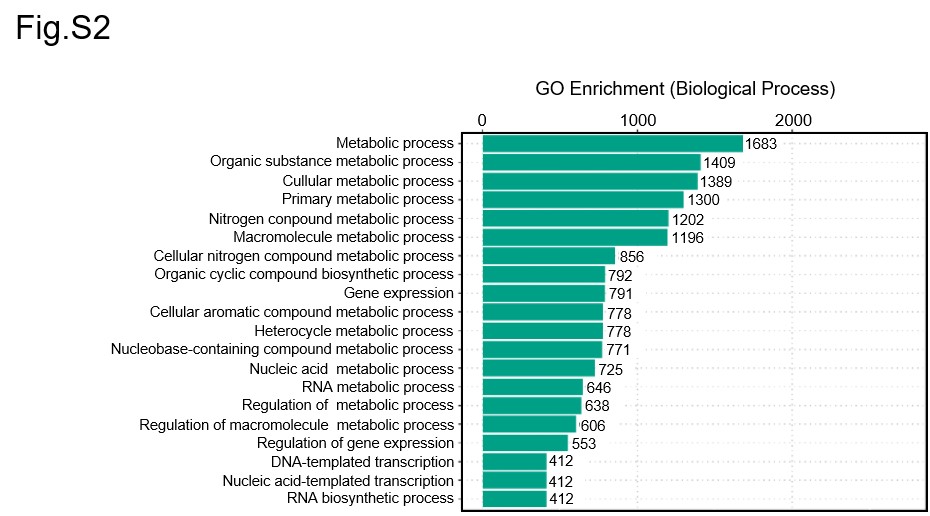

Supplement: Supplementary Figure 2 — Effect of P. plecoglossicida infection on GO Enrichment (Biological Process) in neutrophils of the large yellow croaker. The top 20 GO Enrichment (Biological Process) terms for DEGs. [file Image2.jpeg]
