# Supplementary material for: Pseudomonas plecoglossicida infection induces neutrophil autophagy-driven NETosis in large yellow croaker Larimichthys crocea
Source: Front Immunol. 2024 Dec 23;15:1521080. doi: 10.3389/fimmu.2024.1521080 (PMC11701331; doi:10.3389/fimmu.2024.1521080)
Supplement: Supplementary file 3 [file Table1.docx]

Table S1. Summary of Illumina sequence data.

| Samples | LiC1 | LiC2 | LiC3 | LiE1 | LiE2 | LiE3 |
| --- | --- | --- | --- | --- | --- | --- |
|  |  |  |  |  |  |  |
| Clean Reads Number | 43,327,678 | 43,173,190 | 43,372,706 | 43,293,424 | 40,653,770 | 43,276,572 |
|  |  |  |  |  |  |  |
| Clean Reads ≥ Q30 (%) | 96.7 | 97.4 | 96.2 | 96.2 | 96.1 | 96.0 |
|  |  |  |  |  |  |  |
| GC Content (%) | 46.6 | 46.7 | 46.3 | 47.6 | 47.7 | 47.6 |
|  |  |  |  |  |  |  |

Table S2. Reads mapping information.

| Samples | LiC1 | LiC2 | LiC3 | LiE1 | LiE2 | LiE3 |
| --- | --- | --- | --- | --- | --- | --- |
|  |  |  |  |  |  |  |
| Total (Clean) Reads Number | 43,327,678 | 43,173,190 | 43,372,706 | 43,293,424 | 40,653,770 | 43,276,572 |
|  |  |  |  |  |  |  |
| Total Map (%) | 92.6 | 93.2 | 92.7 | 94.7 | 92.4 | 94.7 |
|  |  |  |  |  |  |  |
| Unique Map (%) | 73.8 | 74.4 | 74 | 80.6 | 74.8 | 82.6 |
|  |  |  |  |  |  |  |
| Multi Map (%) | 18.7 | 18.8 | 18.7 | 14.1 | 17.6 | 12.2 |
|  |  |  |  |  |  |  |

Table S3. Primers used for qPCR.

| Molecule | Primer sequence | GeneBank Accession |
| --- | --- | --- |
| lc3-F | GGTCATCATTGAACGGTACGAGAGG | XM_010741367.3 |
| lc3-R | GTCCGAACATCTCCTGCGAAGC |  |
| bcl2l1-F | TCTGTGCGTCATCATCATACTTGGC | XM_010743692.3 |
| bcl2l1-R | GTCGGTGCTGCTCCATCTGTTG |  |
| dapk-F | GCTGTGATGTTGTGAGGAGAGTGAG | XM_027281486.1 |
| dapk-R | TCTGCCGCTTCTTGATGAACTTGG |  |
| hmgb1-F | AGACGAGGAGGACGACGATGATG | XM_010750658.3 |
| hmgb1-R | CCGCCTGGGTTGGTTCTTGTG |  |
| atg13-F | GGAAGGAAGGCGGAGTTGTGTTG | XM_019258796.2 |
| atg13-R | GAGGAGAGTCTGGAGGCTGTACC |  |
| actin1-F | GGTTACGAAGAGTGGCTCCTCAATG | XM_027275000.1 |
| actin1-R | CCTGTGCGATGGCTGCGATC |  |
| actin4-F | GCCTCGCCTTCAACGCTCTG | XM_027279895.1 |
| actin4-R | CAGTCTTCTCTTGGGTTCGGTTCTC |  |
| rock1-F | CGGATTGTCTTCTGGATGGCTTGG | XM_010729221.3 |
| rock1-R | GAGGTAGCGGTCATCTTGGAATGC |  |
| rock2-F | CTTGTGGCAGCGGCAGTGAG | XM_027274956.1 |
| rock2-R | AGCAGCAGCAGCAGCAACAG |  |
| myosin-9-F | GACGCAAGAGCAGGCAGAGTTC | XM_019269527.2 |
| myosin-9-R | CAGAGCGAGGATACCAGGAGGAC |  |
| cdc25b-F | ACCACGAGGACTTCAAGGAGGAC | XM_010754317.3 |
| cdc25b-R | GTGTGCGGTCTGCTGGATGTC |  |
| mcm2-F | TAAGATGAACGACGCAGACAGAACG | XM_019272689.2 |
| mcm2-R | GCCACGCCTCCTTCCTTGTTG |  |
| mcm7-F | CGATTACATCACCGCTGCCTACG | XM_019260362.2 |
| mcm7-R | TTGTCGGCTTGTAGTGAATCCTTGG |  |
| cdk1-F | GTCAGCATTCCAGCGGCAGTC | XM_010752427.3 |
| cdk1-R | TCAGGACATCCAGGAGCCTTACG |  |
| ccne2-F | GTGAGCGGCGGATATAAGGATTGG | XM_010756517.3 |
| ccne2-R | TGTTTCTTGGCAGCAGGTTGGAG |  |
| eif4b-F | GGCGGCGTCAGCTAAGAAGAAG | XM_010753674.3 |
| eif4b-R | GCAGGATGGAGCGGTCAATGG |  |
| rps6-F | TGAGGCAGTACGTCGTGAGGAG | XM_019267468.2 |
| rps6-R | CACTTCTGGCTGGACTCTGACTTG |  |
| tsc1-F | GAGGAAGTGGTGAAGCCGATGC | XM_010736764.3 |
| tsc1-R | AGTTCTGCCGTCTGGAGGTCTG |  |
| eif4e-F | ACTCGGCTTCGGCTGTGATTATTG | XM_010744868.3 |
| eif4e-R | CGCTCTTGCTGGATGTGTCGTC |  |
| s6k1-F | AGGTCCACACGGCAACAACAAC | XM_010744713.3 |
| s6k1-R | AACTCCACCTCCTCCGTCTGAAC |  |
| β-actin-F | GACCTGACAGACTACCTCATG | XM_027284923.1 |
| β-actin-R | AGTTGAAGGTGGTCTCGTGGA |  |
